# Molecular Dynamics Simulations of Interactions between Human Telomeric i-Motif Deoxyribonucleic Acid and Functionalized Graphene

**DOI:** 10.1021/acs.jpcb.2c04327

**Published:** 2022-08-29

**Authors:** Tomasz Panczyk, Jolanta Nieszporek, Krzysztof Nieszporek

**Affiliations:** † Institute of Catalysis and Surface Chemistry, Polish Academy of Sciences, ul. Niezapominajek 8, Cracow 30239, Poland; ‡ Department of Analytical Chemistry, Institute of Chemical Sciences, Faculty of Chemistry, Maria Curie-Sklodowska University, pl. Maria Curie-Sklodowska 3, Lublin 20031, Poland; § Department of Theoretical Chemistry, Institute of Chemical Sciences, Faculty of Chemistry, Maria Curie-Sklodowska University, pl. Maria Curie-Sklodowska 3, Lublin 20031, Poland

## Abstract

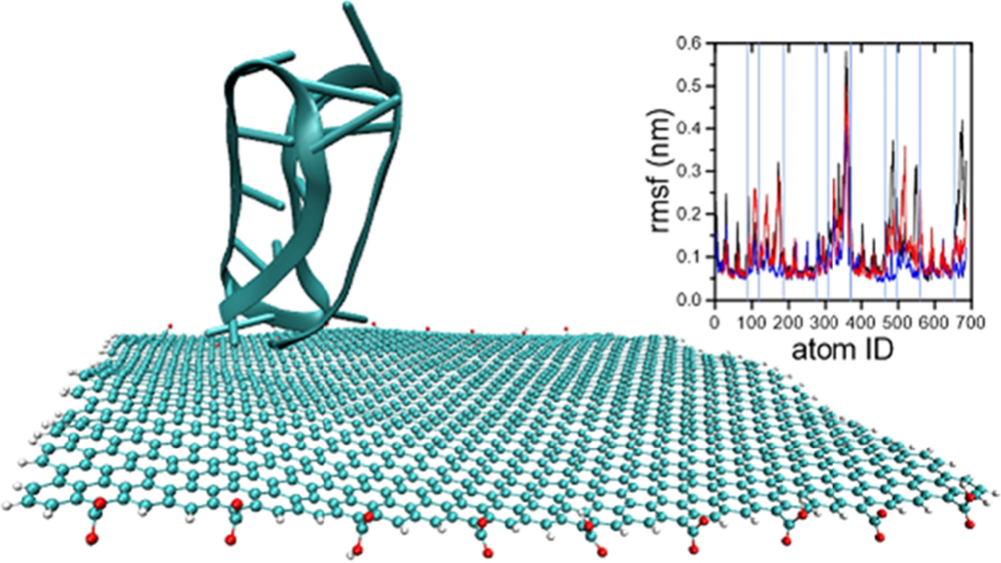

The work deals with molecular dynamics (MD) simulations of protonated, human telomeric i-motif deoxyribonucleic acid (DNA) with functionalized graphene. We studied three different graphene sheets: unmodified graphene with hydrogen atoms attached to their edges and two functionalized ones. The functionalization of graphene edge consists in attaching partially protonated or dissociated amine and carboxyl groups. We found that in all cases the protonated i-motif adsorbs strongly on the graphene surface. The biased MD simulations showed that the work necessary to drag the i-motif out from amine-doped graphene is about twice larger than that in other cases. In general, the system i-motif/amine-doped graphene stands out from the rest, e.g., in this case, the i-motif adsorbs its side with 3′ and 5′ ends oriented in the opposite to surface direction. In other cases, the DNA fragment is adsorbed to graphene by 3′ and 5′ ends. In all cases, the adsorption on graphene influences the i-motif internal structure by changing the distances between i-motif strands as well as stretching or shortening the DNA chain, but only in the case of amine-doped graphene the adsorption affects internal H-bonds formed between nucleotides inside the i-motif structure.

## 1 Introduction

Deoxyribonucleic acid (DNA) is the main building block of the cell nucleus. It contains information necessary for the reproduction, growth, and functioning of living organisms. In the sequence of nucleotides, DNA has genetic instructions with information necessary for the synthesis of all proteins. Moreover, each strand of DNA in the double helix can serve as a pattern for duplicating itself. DNA molecules are very long and are packed into more complex structures called chromosomes. An eukaryotic, i.e., human chromosome, is terminated by a specific sequence of bases called telomere which is normally folded at its very end into a loop. Inside chromosomes, DNA is coiled around histones mainly in a double-helix form. Moreover, a wide spatial versatility of DNA has been observed, like A-DNA, B-DNA, or Z-DNA in addition to the typical Watson–Crick duplex DNA. Also, a number of alternative structures including four-stranded G-quadruplex and intercalated i-motif DNA have been found.^1,2^

In the region of telomeric DNA composed of (TTAGGG/CCCTAA)_*n*_ sequences, an individual DNA strand is able to form a quadruplex structure. When the DNA region is cytosine-rich it may fold into four-stranded, i-motif structure. It has been discovered that a telomeric i-motif can occur in vivo and plays an important role in the control of gene expression.^3−5^

It is commonly assumed that the i-motif structure is stabilized at acidic pH. The stabilizing effect enhances the presence of monovalent ions like Ag^+^, Na^+^, or K^+^ in solution. In addition, a few nanosized carbon materials can work as a stabilizer of the spatial i-motif structure.^6−9^ Due to its adsorption properties and sensitivity to pH, the i-motif can be considered as a bioconjugation factor^8^ and biosensor component.

The interaction of DNA structures with carbon nanomaterials has gained increasing interest in the literature in the past few years. It concerns the interactions of carbon nanomaterials (CNMs) with different structures of DNA, e.g., single-stranded, duplex, triplex, and quadruplex ones. As an example, it has been observed that single- and double-stranded nucleic acids can adsorb onto carbon nanotubes. Single-stranded DNA can helically wrap around a single-walled nanotube. In the case of double-stranded DNA, its adsorption at the nanotube is weak. Nevertheless, it was reported that double-stranded DNA wrapped around nanotube is protected from enzymatic cleavage.^10,11^ Almost unlimited possibilities of the practical use of DNA interactions with nanotubes have been proposed.^12^

In addition to carbon nanotubes, graphene is also the subject of intense research. Most often it relates to graphene oxide which is considered as the component of biosensors, drugs, and gene nanocarriers. Similar to carbon nanotubes, single-stranded DNA shows stronger adsorption affinity to graphene. It has been found that cations, pH, and organic solvent influence DNA binding to graphene oxide.^13^ The binding affinity of nucleic acid to graphene oxide is dependent on the presence of a single-stranded region.^14^ The adsorption/desorption event of nucleic acid on nanomaterials is one of the key steps for effective DNA and RNA isolation.^13,15,16^

Moreover, in the case of graphene oxide, it has been observed that the binding affinity depends on the DNA sequence.^17^ Also, double-stranded DNA can adsorb at oxidized graphene but in high ionic strength solutions. One of the most interesting applications of graphene-DNA systems is molecular computing.^18^ Such biomolecular systems can play a role in logic gates.^19^ Due to the outstanding physical characteristics for DNA sequencing, graphene can also be considered as a key component of modern virus tests.^20^

The aim of this study is a detailed analysis of the i-motif interaction and adsorption on an edge functionalized graphene layer. We will investigate how the functionalization style influences the i-motif adsorption and how adsorption affects the i-motif spatial structure. In addition, we will carefully examine the physical properties of the investigated systems.

## 2 Calculation Methods

The studies were carried out using Gromacs 2020 simulation suite^21^ and employing generalized amber force field (GAFF)^22^ for the functionalized graphene sheet and parm99 Amber force field with bsc1 modifications for the ssDNA fragment.^23^ The structure of the i-motif considered in this study has been obtained by modifying the structure published by Phan et al.^24,25^ (pdb ID 1EL2). The topology of the protonated i-motif has been built applying AmberTools 20 package^26^ and ACPYPE script.^27^

**Figure 1:**
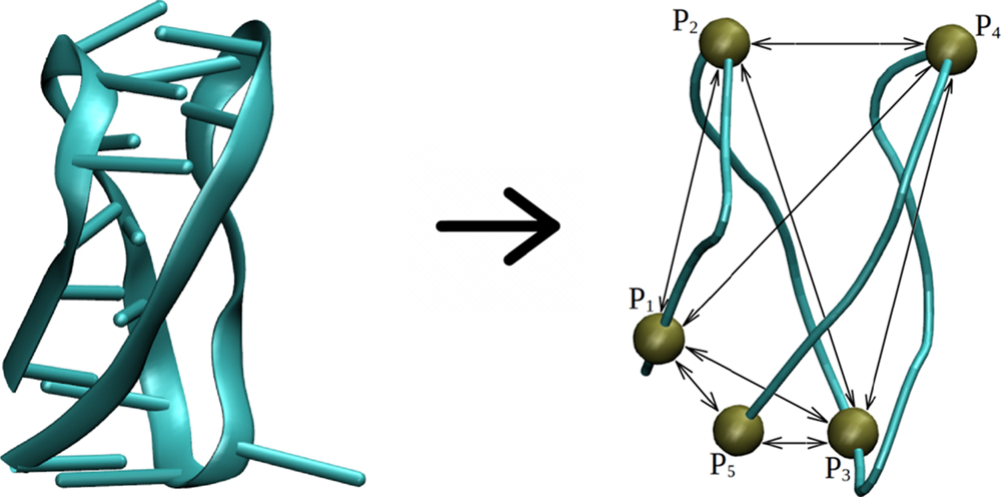
Protonated i-motif with indicated P1–P5 phosphorus atoms used to investigate its spatial configuration during molecular dynamics (MD) simulations. P1 is the phosphorus atom from i-motif 5′-end, whereas P5 is the one from 3′ end.

During simulations, we investigated the spatial configuration of the i-motif by monitoring the distances between selected phosphorus atoms from the backbone located at the characteristic points of the structure, as shown in Figure 1.

**Figure 2:**
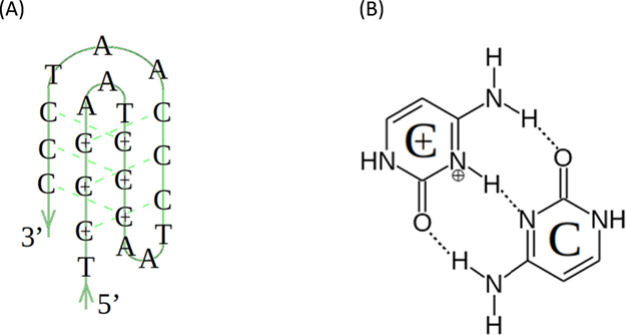
Schematic representation of the i-motif structure studied in this work: (A) spatial scheme of the DNA fragment in the form of the i-motif consisting of the sequence (CCCTAA)_2_C^+^C^+^C^+^TAAC^+^C^+^C^+^T, (B) three hydrogen bonds formed between protonated C^+^ and unprotonated cytosines C within the folded, four-stranded i-motif structure.

It is commonly assumed that the i-motif is stable at acidic pH, i.e., when a part of cytosines from the DNA strand is protonated.^24,25,28^ Thus, we studied the structure in which half of the cytosines are protonated. The sequence of nucleobases in the DNA chain is then (CCCTAA)_2_C^+^C^+^C^+^TAAC^+^C^+^C^+^T (see Figure 2).

**Figure 3:**
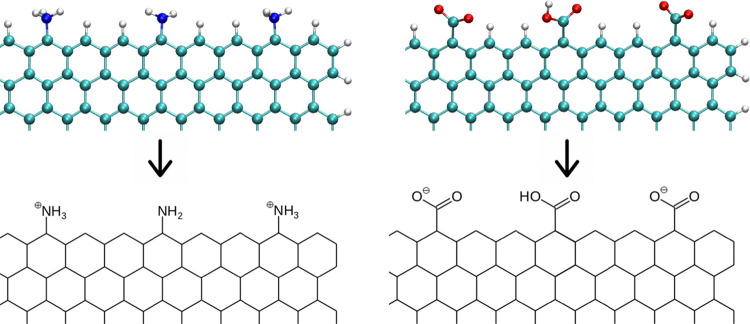
Illustration of the applied functionalization of graphene sheets. Colors in the top part of the figure represent atoms: carbon – green, hydrogen – gray, nitrogen – blue, oxygen – red. Total charge of amine-doped graphene, GR-NH_2_, is +9, whereas, in the case of carboxyl-doped one, GR-COOH, is −9.

The functionalization of graphene sheets assumed attaching amine and carboxyl groups to their edges. Half of them are in charged form: amine groups are protonated and carboxyl groups are dissociated. These functional groups are present in a lot of biological systems. Because of the large number of atoms in graphene sheets applied in the present studies (circa 2000 atoms, 70 nm × 70 nm sheet sizes), only their characteristic fragments were used to build topologies. These characteristic fragments included about 50 atoms and were about 9 nm × 9 nm in sizes. This procedure is due to the fact that R.E.D. Server^29^ used to determine the atomic charges accepts only up to 250 atoms. Thus, atomic charges for atoms in a sheet have been obtained using the RESP-A1 (HF/6-31G*) charge model and the Gaussian 09 quantum mechanics program. Next, the information obtained from the R.E.D. Server has been applied to the whole graphene sheets by manual edition of MOL2 files. Finally, using ACPYPE, we have built the force field topology according to the GAFF parameterization. Graphene sheets are almost rectangular and only two opposite edges were functionalized (Figure 3). In the case of amide-doped graphene (GR-NH_2_), its total charge is +9, whereas for the carboxyl-doped one (GR-COOH), the total charge of the sheet is −9. Carbon atoms at the graphene edge are saturated with hydrogen atoms.

Simulations were carried out in the *NPT* ensemble in the cubic box with sizes approximately 11 nm × 11 nm × 11 nm. Boxes were composed of one i-motif and one graphene sheet, about 40,000 tip3p water molecules and a dozen of Na^+^ and Cl^–^ ions to imitate 0.145 mol/dm^3^ ionic strength.

The temperature of simulation systems was maintained at 310 K using a Nose–Hoover thermostat. Periodic boundary conditions were applied. For long-range electrostatic interactions, the smooth particle-mesh Ewald algorithm was used. Cutoff distances for Lennard–Jones and Columbic interactions were set to 1.2 nm. All particles were placed randomly in the simulation box. Equations of motion were integrated using the Leap-Frog algorithm with the time step of 2 fs. Each simulation was equilibrated for 10 ps. Based on our previous experience in studying the interaction of DNA with carbon nanotubes, e.g., in ref (30), we decided to set the simulation times to 45 ns. In scientific literature, papers on similar topics can be found in which authors conducted simulations for much longer times, e.g., 100 ns^31^ or even 1000 ns^32^ but we think that in our case it is possible to simulate a little shorter. This is confirmed by the values of parameters monitored during the simulation, e.g., the root of mean squared displacement (RMSD) of the i-motif from its initial state. As we will show in the work later, after a maximum of several nanoseconds, the values of different parameters monitored during simulations shortly reach an approximately constant value.

## 3 Results and Discussion

**Figure 4:**
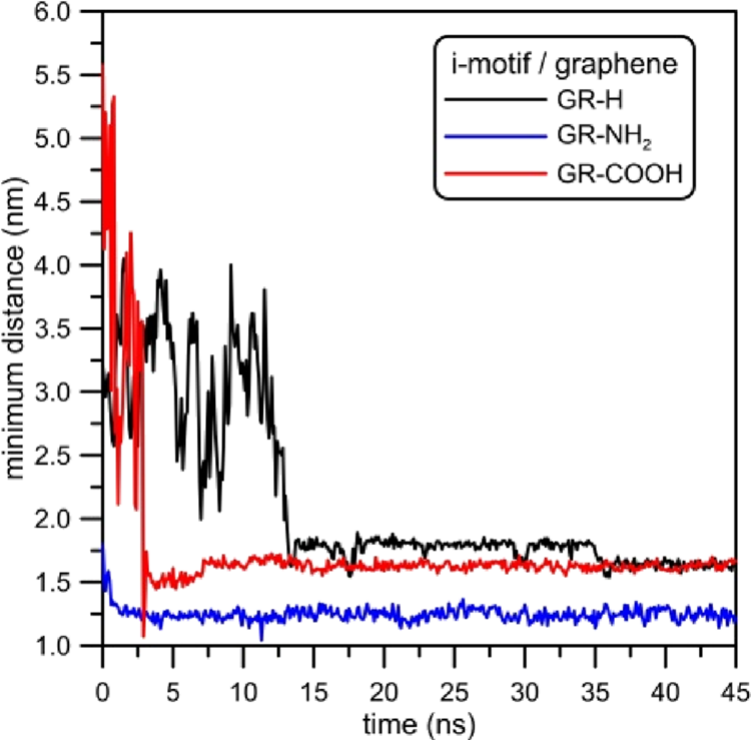
Minimum distances between the COM of the i-motif and a carbon atom from functionalized graphene plotted as the function of simulation time.

The studies were carried out for three systems that differed in the method of graphene functionalization. Instead of amine- and carboxyl-doped graphene, GR-NH_2_ and GR-COOH, which are positively and negatively charged, respectively, the charge of graphene sheet where functionalization has not been applied is neutral. It could be assumed that the electric charge of graphene is one of the most important factors which influences the interactions with the protonated, i-motif DNA fragment. This thesis confirms the minimum distance between the center of mass (COM) of the i-motif structure and the closest atom from the graphene surface, calculated for investigated systems. Figure 4 shows that depending on the type of functionalization the equilibrium distances between the i-motif and graphene are different. Moreover, it can be seen that the time in which the i-motif molecule approaches graphene is the shortest for the amine-dosed one. The minimum distance between GR-NH_2_ and COM of the i-motif is reached at about 1 ns. In the case of the system with GR-COOH, this time is 7 ns. For the simulation system which includes graphene without functionalization, GR-H, the process in which the i-motif approaches the graphene surface is the most time consuming, as it takes approximately 15 ns. Also, the minimum distance between these two structures depends on the type of graphene functionalization. In the case of GR-H and GR-COOH, when the i-motif reaches the graphene surface, the minimum distances between these two structures are initially a bit different. The distance of the COM of the i-motif and GR-H is ca. 1.8 nm, whereas the distance from the GR-COOH surface is about 1.6 nm. After 35.5 ns of simulations, the i-motif molecules get closer to GR-H and reach almost the same position as in the i-motif/GR-COOH. It is worth noting that the fluctuations of distance for both pairs are similar but for the system with GR-NH_2_ they are a little bigger. After reaching the equilibrium configuration, the minimum distances of i-motif/GR-H and i-motif/GR-COOH vary in the range of ca. 0.25 nm, whereas the minimum distance between the DNA fragment and GR-NH_2_ varies in the range of ca. 0.3 nm.

**Figure 5:**
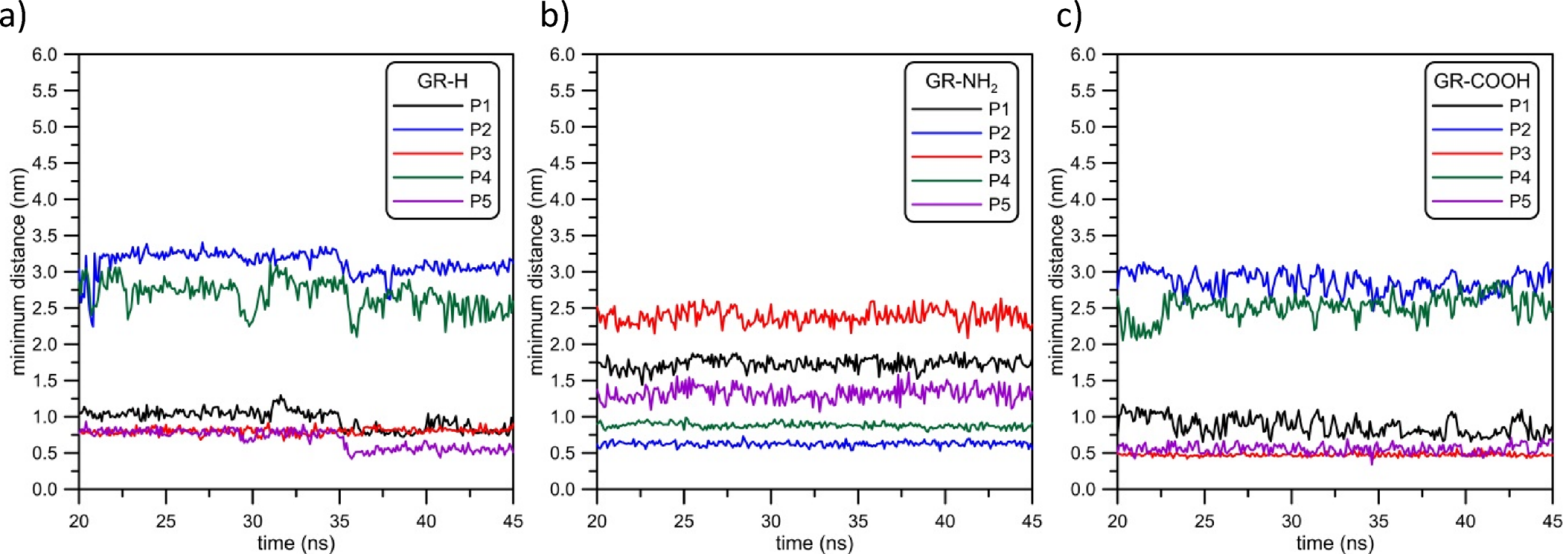
Minimum distances between GR-H, GR-NH_2_, and GR-COOH surfaces and P1–P5 phosphorus atoms within the i-motif structure (see Figure 1).

Because the starting configuration of simulated systems was generated in a random way, the time consumed for the DNA fragment to approach the graphene plane says little about the physical affinity between these two structures. Nevertheless, it is clearly seen that, in comparison to other systems, the amine-doped graphene interacts with the i-motif in a different way. Detailed examination of the positions of P1–P5 points shown in Figure 1 will provide information about the mutual arrangement of the i-motif and graphene structures. Figure 5 shows the distances of those phosphorus atoms from the graphene surface. For the sake of clarity, we presented the data for time simulation range of 20 to 50 ns, i.e., we skipped the times when the i-motif has not yet reached the graphene surface.

**Figure 6:**
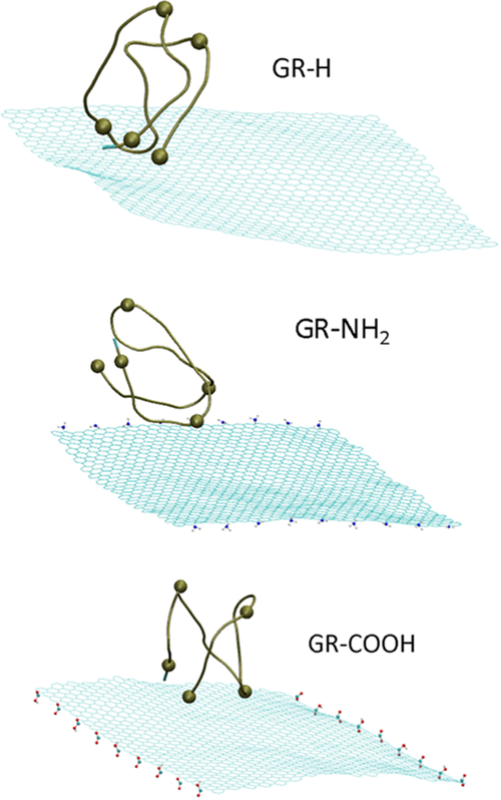
Simulation snapshots recorded at 40 ns showing the i-motif adsorbed at graphene surfaces. For the clarity of presentation water molecules and ions are hidden.

For the record, P1, P3, and P5 phosphorus atoms are located on the i-motif’s side with 3′ and 5′ ends, whereas P2 and P4 are on its opposite side. Figure 5 shows that all P1–P5 distances from the graphene plane recorded for GR-H and GR-COOH are similar for these two systems. The closest i-motif COM distance appears for the GR-NH_2_ system, as showed in the part (b) of Figure 5. It can also be seen that the average distance of the P3 atom from the graphene surface (red lines in Figure 5) is about 2.5 nm. For systems composed of GR-H and GR-COOH, they are about 0.7 and 0.5 nm, respectively. Simultaneously, the minimum distance of, e.g., P2 phosphorus atom (blue lines in Figure 5) from the GR-NH_2_ surface is approximately 0.6 nm, whereas the analogous distances from GR-H and GR-COOH are approximately 3 and 2.7 nm. Further comparison of the distances presented in Figure 5 allows to conclude that the i-motif orientation at graphene surfaces is different: in the case of GR-H and GR-COOH, it is oriented with its 3′ and 5′ ends toward the graphene surface, whereas the i-motif structure adsorbed at GR-NH_2_ is oriented “upside down,” i.e., the DNA fragment is adsorbed to graphene with the side containing P2 and P4 atoms. Moreover, the fluctuations of minimum distances shown in Figure 5 for the system with amine-doped graphene are the smallest. It could mean that the i-motif/GR-NH_2_ dimer is the most stable among the considered systems. Figure 6 shows the snapshots from simulations trajectories recorded at 40 ns.

As seen in Figure 6, the protonated i-motif binds to the graphene surface in different ways. These differences are caused by the functionalization type of the graphene edge. A similar relationship has been found recently.^30^ When studying i-motif interactions with single-walled carbon nanotubes, we also observed its different orientation with respect to the nanotube surface. While using the same terminology, the arrangement of the i-motif in the case of adsorption on GR-NH_2_ can be called as “top” orientation, whereas in other cases we have the “bottom” orientation.

**Figure 7:**
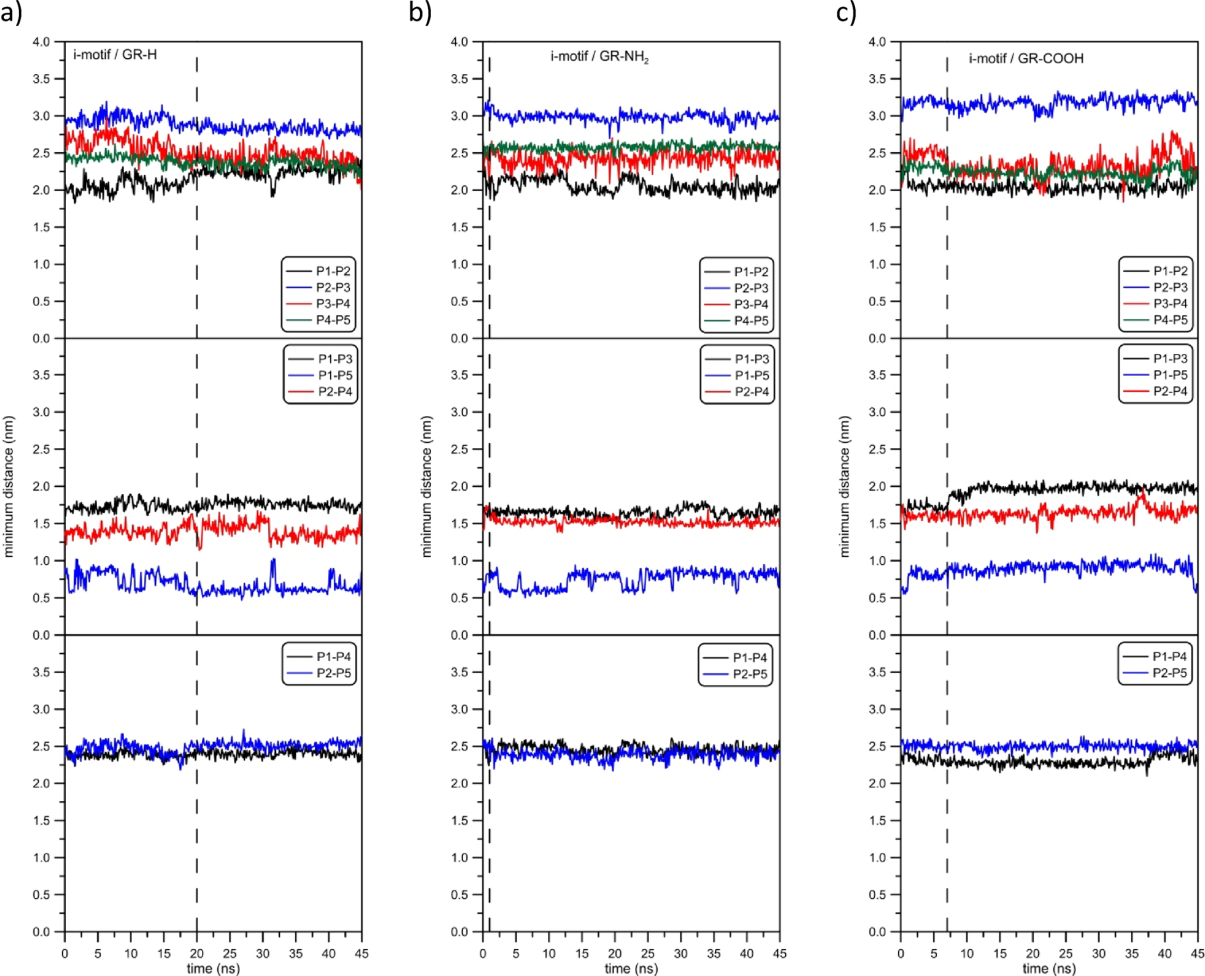
Quantitative analysis of the effect of i-motif adsorption at the modified graphene plane on its structure: parts (a–c) relate to their adsorption at GR-H, GR-NH_2_, and GR-COOH graphene sheets. Vertical, dashed lines relate to times when the i-motif reached the graphene surface.

One of the most interesting effects is the influence of graphene plane on the i-motif structure. The quantitative analysis of such an effect can be done through the investigation of mutual distances between P1, P2, P3, P4, and P5 phosphorus atoms. Figure 7 shows the comparison of these values for all investigated systems. For the Reader’s convenience, in Table 1 we inserted the values of average as well as the standard deviations of mutual distances between selected phosphorus atoms after i-motif adsorption occurrence.

**Table 1:** Average Values as Well as Standard Deviations of Distances between Selected Phosphorus Atoms from the i-Motif Backbone after Its Adsorption on the Graphene Surface

P atoms	GR-H		GR-NH_2_		GR-COOH	
	average (nm)	st. dev. (nm)	average (nm)	st. dev. (nm)	average (nm)	st. dev. (nm)
P1–P2	2.24	0.08	2.06	0.09	2.04	0.07
P2–P3	2.82	0.06	2.98	0.06	3.18	0.08
P3–P4	2.44	0.11	2.42	0.09	2.31	0.15
P4–P5	2.35	0.06	2.58	0.05	2.22	0.07
P1–P3	1.76	0.05	1.64	0.06	1.97	0.06
P1–P5	0.62	0.09	0.75	0.11	0.91	0.07
P2–P4	1.39	0.10	1.52	0.04	1.64	0.08
P1–P4	2.40	0.05	2.46	0.07	2.29	0.07
P2–P5	2.52	0.05	2.38	0.07	2.50	0.05

Even the cursory analysis of Figure 7 as well as the data collected in Table 1 show that the i-motif structure changes due to adsorption interactions. While using the i-motif and GR-H as the reference system, it can be seen that the length of the DNA chain changes, e.g., the distance between P4 and P5 phosphorus atoms becomes larger when the i-motif is adsorbed at GR-NH_2_ and decreases when it is adsorbed at GR-COOH (Figure 7b,c). This difference is about 0.25 nm. A greater or lesser chain stretching/shortening effect can also be seen for other DNA fragments (please compare top parts of Figure 7a–c which show the distances between neighboring P1, P2, P3, P4, and P5 atoms). In turn, the middle parts of Figure 7 show the effect of the i-motif adsorption at graphene planes on its construction consistency, i.e., the distances between selected P atoms which show how tightly i-motif strands are related to each other. Again, we can observe visible differences between studied systems. First, the fluctuations of distances monitored by P1–P3, P1–P5, and P2–P4 are the highest in the case of GR-H. Second, the distance between P2 and P4 phosphorus atoms is the lowest when the i-motif is adsorbed at GR-NH_2_. Thirdly, the distance between P1 and P3 is the highest for the system with GR-COOH and exceeds the distances in other systems by more than 0.5 nm. The bottom parts of Figure 7 show P1–P4 and P2–P5 distances which represent the diagonal distances inside the i-motif structure. In this case, the most noticeable effect of adsorption occurs for carboxyl-doped graphene.

**Figure 8:**
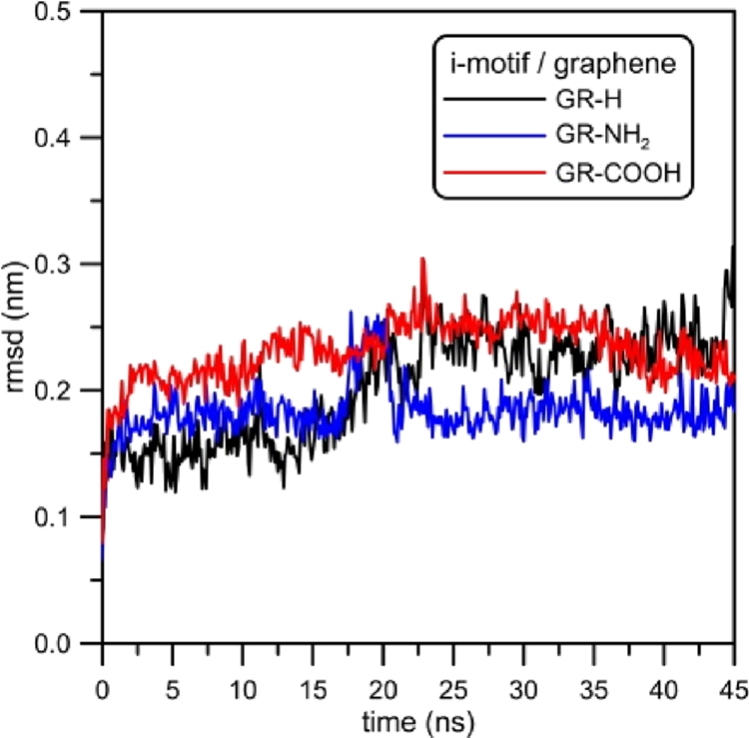
RMSD plotted as the function of time for the i-motif in the analyzed systems. Reference states are the structures of i-motifs at the zeroth timestep.

A valuable tool to investigate how the adsorption of i-motif on the graphene surface affects its internal structure can be the RMSD from the initial states. Figure 8 shows the RMSD plotted as a function of time. The RMSD plots were determined for all i-motif atoms and were normalized, i.e., the translation of COM was subtracted and the i-motif structure was rotated to adjust a given state with the reference, initial one.

**Figure 9:**
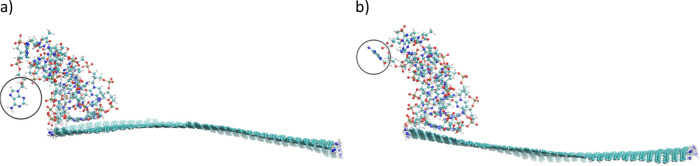
Snapshots from the i-motif/GR-NH_2_ simulation trajectory taken at approx. (a) 18 ns and (b) 23 ns. Nucleoside of interest is marked by circle.

For the record, times necessary for the i-motif to reach graphene surfaces were as follows: 15, 1, and 7 ns for GR-H, GR-NH_2_, and GR-COOH, respectively (see Figure 4). The change of RMSD is not large (RMSD values about 0.30) and can also be interpreted as effects due to thermal fluctuations. Nevertheless, the analysis of RMSD curves presented in Figure 8 makes it possible to state that the contact between the DNA fragment and graphene sheet results in the change of the i-motif spatial structure. Starting with the system containing GR-H, a visible growth in RMSD at times approx. 15 ns can be seen. On an average, in this case, the RMSD increases from 0.15 nm to about 0.23 nm. Analogously, when the i-motif adsorbs at GR-NH_2_ or GR-COOH, the corresponding RMSD functions also increase but the changes are not as clear as for the system with a nonfunctionalized graphene sheet, they do not exceed 0.05 nm. In the case of the i-motif/GR-NH_2_ system in the range from 15 to 25 ns, a momentary jump of the RMSD function is observed. Responsible for that is the nucleoside which includes nucleobase adenine located on the arc of the helix where 3′ and 5′-ends are (see Figure 1, bottom left part of the picture). This is shown in Figure 9 where two snapshots from the simulation trajectory of the i-motif/GR-NH_2_ system are presented. It is worth noting that this effect does not occur when the i-motif is adsorbed at GR-H or GR-COOH—in such cases the i-motif is adsorbed on graphene toward its 3′ and 5′-ends and that nucleoside with adenine is flatly adsorbed on the graphene surface.

Therefore, the jump observed in the RMSD function in the range of 15 to 25 ns determined for the i-motif/GR-NH_2_ system is caused probably by temporary flaps of the nucleoside with adenine which is nonbonded to another nucleobase like thymine.

**Figure 10:**
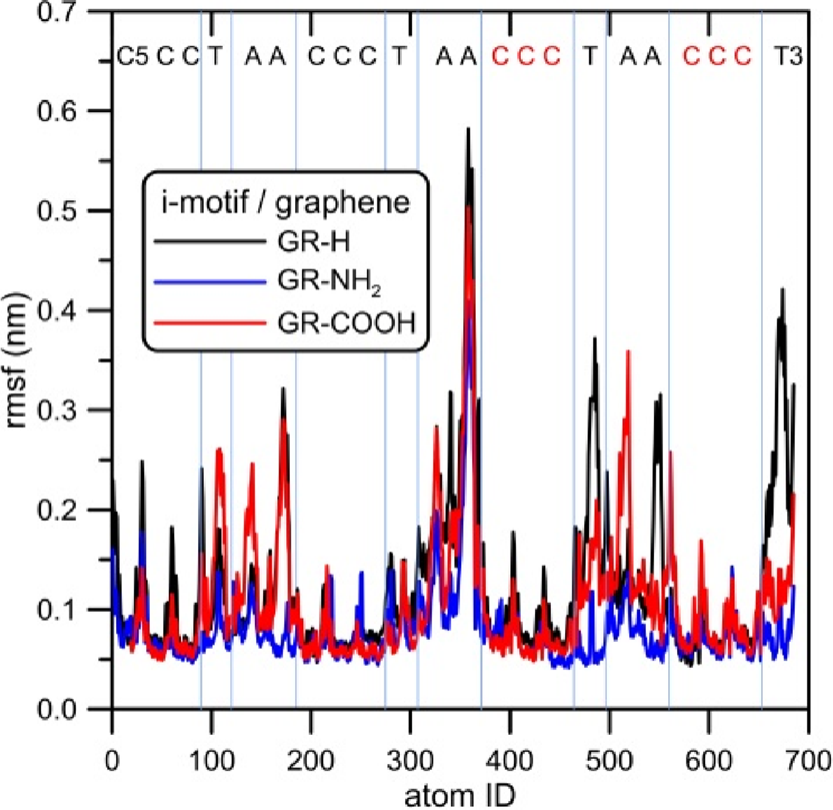
RMSF of atomic positions within the adsorbed i-motif structure. Letters on the top show which residues the given atomic numbers belong to. Red C letters denote the protonated cytosines.

Figure 10 shows fluctuations of atomic positions after i-motif adsorption on the graphene surface. The plot of the root of mean squared fluctuation (RMSF) informs about the mobility of individual atoms or residues (as indicated on the top part of the figure). We can conclude that the least mobile are C:C+ pairs and the range of their fluctuations from the reference state is actually the same. This means that the adsorption on graphene does not affect strongly bonded semiprotonated cytosine pairs. For all studied systems, the most mobile are adenine residues localized in the region of the P3 atom (atom IDs about 350, see also Figure 1). The difference appears for 3′ thymine – the range of fluctuations is quite large when it is adsorbed on GR-H, whereas in other cases they are actually the same. The differences are seen for thymine and adenines localized in the body of the i-motif because these bases are not stabilized by hydrogen bonds. In the range of atom IDs 89 to 185, when the i-motif is adsorbed on GR-COOH, we can observe significant flexibility of thymine and adenines. In this range of the i-motif structure, we can also notice the stabilizing effect of adsorption on GR-NH_2_, the fluctuations of thymine and cytosine atomic positions are very low. The stabilization of the i-motif structure adsorbed on GR-NH_2_ is also seen in the upstream part of the DNA strand. In the atom ID range of 371–560, when the i-motif is adsorbed on GR-NH_2_, the mobility of thymine and adenines is very low. In this part of the i-motif structure, we can observe visible flexibility of these nucleobases adsorbed on GR-H or GR-COOH.

**Figure 11:**
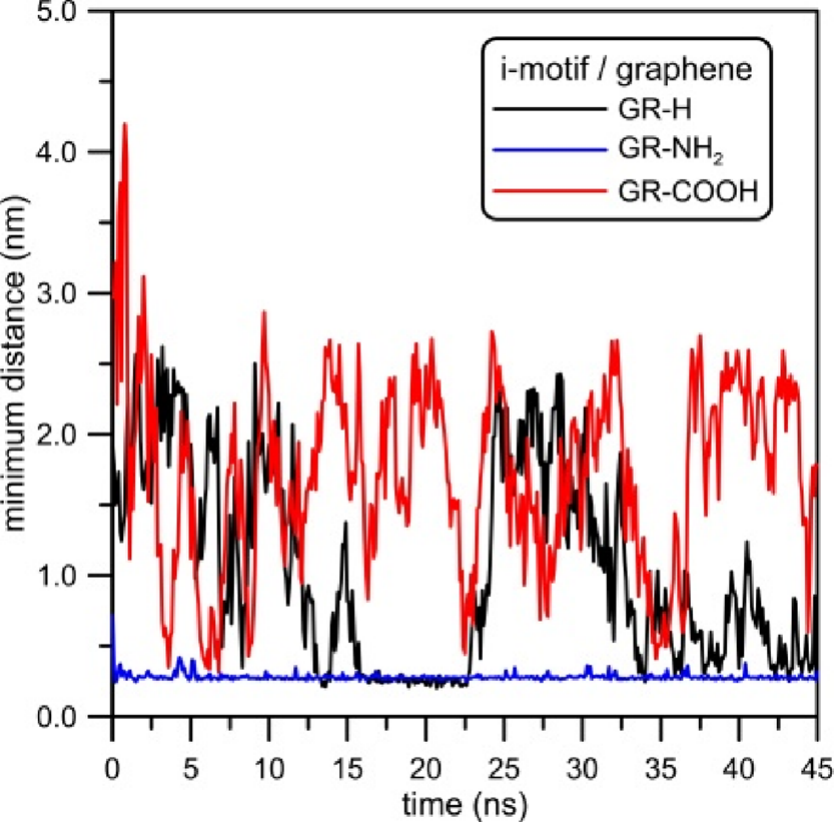
Minimum distances between the i-motif and functionalized graphene edges.

Figure 9 shows that at times when snapshots were taken, the i-motif is adsorbed right on the graphene edge. Thus, to analyze more precisely the position of DNA on the graphene plane we investigated the distances between any closest i-motif atom and an atom from the functionalized graphene edge: hydrogen, nitrogen, or carbon from the carboxyl group for GR-H, GR-NH_2_, and GR-COOH, respectively. Results can be seen in Figure 11.

It can be concluded that in the case of GR-NH_2_, the i-motif structure stays almost at the edge of the graphene plane. The distance between an i-motif atom and a nitrogen one from the amine group is about 0.3 nm. This very close distance is likely due to electrostatic attraction: DNA fragment is in anionic form, its total charge is −15, and a part of amine groups attached to the graphene edge is protonated. In other cases, the adsorbed i-motif structure resides in different areas of the graphene sheet. When it is adsorbed at GR-COOH, the most preferred place is near the central part of the graphene plane (the length of the edges of the graphene sheet is about 7 nm). It is due to the fact that a part of carboxyl groups is in anionic form. Finally, the i-motif structure adsorbed at nonfunctionalized graphene GR-H in some period of simulation trajectory also reaches the graphene edge. This is due to charge distribution between carbon atoms in graphene and hydrogen atoms at its edge. While carbon atoms in the center of the sheet have neutral charge, the carbon atoms near the graphene edges are increasingly negative charged. The total charge of the graphene sheet is balanced by positively charged hydrogen atoms. Thus, when the i-motif comes close enough to the edge of the GR-H sheet, it falls into a potential well where it is attracted by positively charged hydrogen atoms that are on that edge.

**Figure 12:**
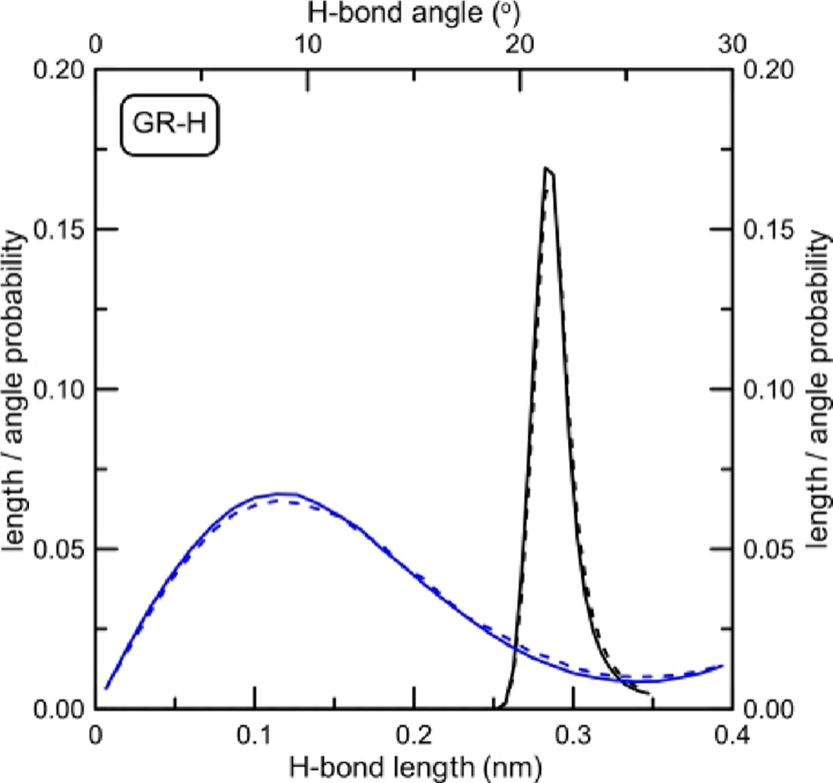
Hydrogen bond angle and distance probability distributions between nucleobases inside the i-motif structure determined for the system with nonfunctionalized graphene. Dashed lines represent the state when the i-motif is not adsorbed at the graphene, whereas solid lines are length and angle distributions for the i-motif adsorbed at GR-H. Dashed lines were calculated for simulation times <13 ns, whereas solid lines were determined for times >15 ns.

The next feature investigated is hydrogen bonds between nucleotides inside the i-motif molecule. It is worth recalling that, in the MD simulations, hydrogen bonds are defined by two geometrical parameters *r* and α. One of them is the distance *r* between two strongly electronegative atoms D and A from hydrogen bond donor D–H and hydrogen bond acceptor A. The second one is the angle α between three atoms D–H···A. The bond becomes stronger when defined in such a way that distance *r* and angle α become smaller. Here, we applied the most commonly used values of H-bond parameters: *r* ≤ 0.35 nm and α ≤ 30°. Thus, when both criteria are fulfilled, the H-bond bridge is formed. Figures 12^13^,14 show the probability distributions of distances and angles of H-bonds created between nucleobases inside the i-motif determined for all investigated systems.

**Figure 13:**
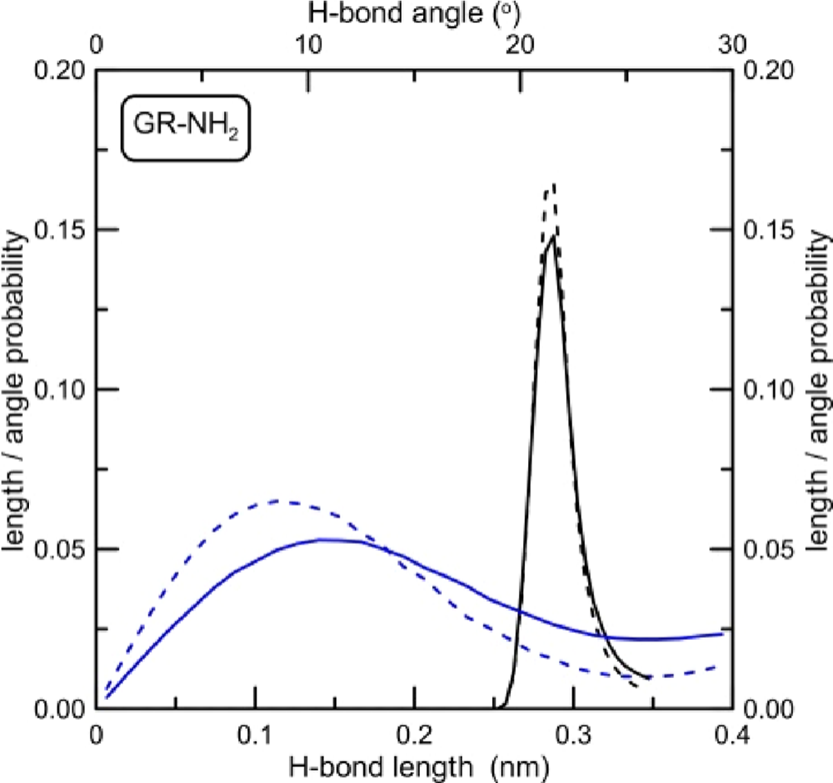
Hydrogen bond angle and distance probability distributions between nucleobases inside the i-motif structure determined for the system with amine-doped graphene. Dashed lines represent the state when the i-motif is not adsorbed at graphene, whereas solid lines are length and angle distributions for the i-motif adsorbed at GR-NH_2_. Dashed lines are exactly the same as in Figure 11, whereas solid lines in Figure 12 were determined for simulation times >1 ns.

**Figure 14:**
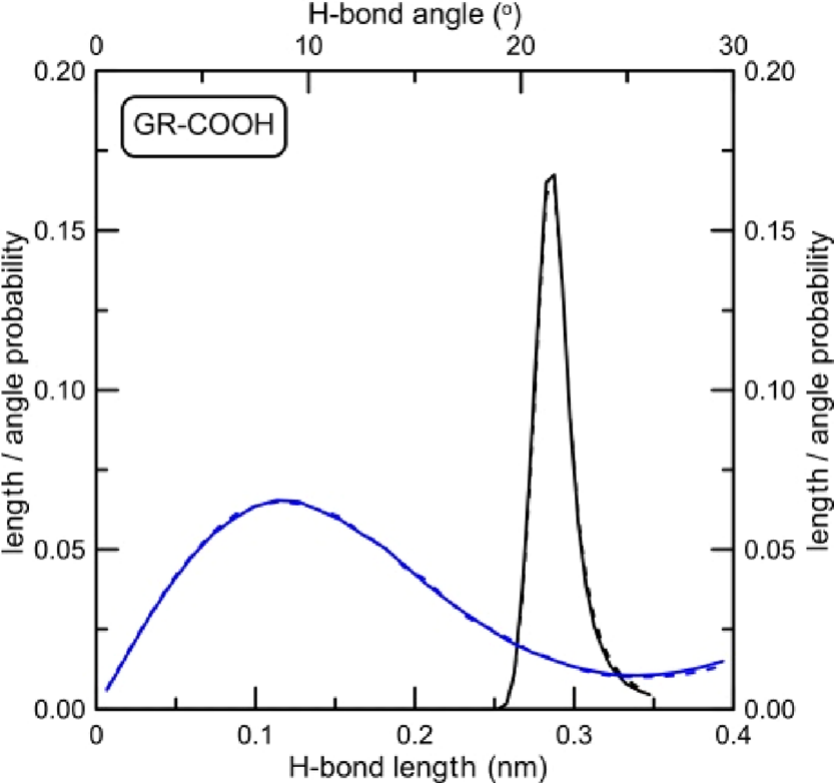
Hydrogen bond angle and distance probability distributions between nucleobases inside the i-motif structure determined for the system with carboxyl-doped graphene. Dashed lines represent the state when the i-motif is not adsorbed at graphene, whereas solid lines are length and angle distributions for the i-motif adsorbed at GR-COOH. Dashed lines are exactly the same as in Figure 11, whereas solid lines in Figure 13 were determined for simulation times >7 ns.

It is worth noting that only in the case of GR-H, we have a relatively long simulation trajectory for the nonadsorbed i-motif at disposal (see Figure 4). In other cases, the times necessary for DNA fragment to reach the graphene plane were quite short. Thus, we used that part of trajectory as the reference state for the i-motif in bulk solution. Of course, the H-bond angle and distance distributions for the state when the i-motif is not adsorbed at the graphene are very similar for all investigated systems. Minor discrepancies from the reference GR-H curves result from a short fragment of the analyzed trajectory (especially in the case of the system with GR-NH_2_).

What we can see from Figures 12–14 is that the adsorption of the DNA fragment at the graphene plane has no visible impact on its internal H-bonds between nucleobases when graphene is nonfunctionalized or is functionalized by carboxyl groups. Distributions shown in Figures 12 and 14 do not change (dashed and solid lines overlap). A completely different situation occurs when the i-motif is adsorbed at the amine-doped graphene GR-NH_2_. While H-bond lengths between i-motif nucleobases remain unchanged, their mutual orientation changes slightly. The most probable H-bond angle changes from about 8° to 11°. Although it is not a big change, the shapes of the angle distribution curves shown in Figure 14 are markedly different. Following Jeffrey’s classification, the decrease of hydrogen bond directionality results in a decrease in bond strength.^33^ Nevertheless, angles change in a relatively narrow range from 0° to 20°. A much more narrow range of changes occurs in the case of H-bond distance distributions. It is always a very narrow peak with the maximum at about 0.29 nm. These values indicate that hydrogen bonds between nucleotides inside the i-motif structure have, in most cases, a strong character with dominant strongly covalent interactions.

In comparison to other investigated systems, we observed that when the i-motif adsorbs at the amine-doped graphene, the fluctuations of distances between its selected phosphorous atoms are the smallest and, simultaneously, the directionality of i-motif internal H-bonds in this system decreases. Thus, it seems that the influence of amine-doped graphene on the stability of the DNA fragment is much more complex. It is worth noting that among the investigated systems, only at GR-NH_2_, the i-motif adsorbs “upside down,” i.e., with its 3′ and 5′ ends on the opposite side to the graphene surface (the so-called “top” orientation^30^). It seems that this effect may mainly be responsible for the different properties of the i-motif/GR-NH_2_ system.

**Figure 15:**
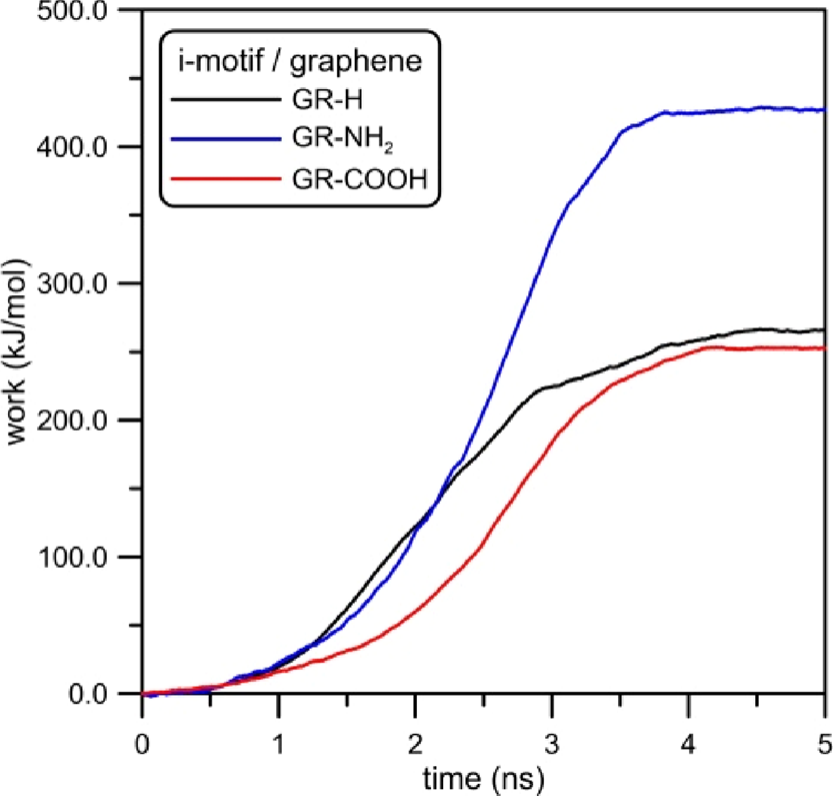
Work done during enforced dragging of the i-motif structure from the graphene surface. Applied pulling velocity was 3.14 × 10^–4^ nm/ps and spring force constant was 30 kJ/mol/nm^2^.

Thus, we can state that the DNA fragment is more strongly adsorbed by amine-doped graphene (see Figure 4). Therefore, we attempt to gather more information about the adsorption energies of the i-motif by using biased MD simulations. The parameter that could approximately describe that adsorption energy is the amount of work necessary to drag the i-motif out of the graphene surface. These analyses were determined by attaching a moving spring to the COM of the i-motif and applying constant velocity pulling. Figure 15 shows the results of such calculations.

It is worth mentioning that the constant velocity dragging can formally be used to determine the free energy.^34^ In such a case, it is necessary to average a lot of trajectories determined from various initial configurations. Unfortunately, such a procedure is numerically complicated even for simple systems. For this reason, the work profiles shown in Figure 15 were obtained only for single runs and they cannot be identified with free energies of i-motif adsorption. It cannot be directly related to the interaction potential between graphene and the i-motif but it can be understood as an upper limit of the free energy of binding of these two species in aqueous solution. This is because the application of Jarzynski equality^34^ ensures that exponential averaging of many work versus distance/time profiles can lead to the exact free energy profile in the limit of infinite number of such trajectories determined. Because we used only a single trajectory, the obtained profiles are probably far from a true free energy of binding. Nevertheless, application of the same settings during the enforced detachments allows us to compare properties of these three systems and draw reliable conclusions about their stability.

Nevertheless, the analysis of Figure 15 can provide some information about the processes occurring during the dragging.

First of all, we can see a huge difference between the work necessary to remove i-motif from various functionalized graphene surfaces. In the case of GR-NH_2_, the required work is approx. twice larger than in the case of GR-H and GR-COOH. It is about 427 kJ/mol, whereas the work necessary to drag i-motif out from GR-H and GR-COOH is 265 and 252 kJ/mol, respectively. Such results are consistent with our previous observations and confirm the significant role of electrostatics interactions in the i-motif/GR-NH_2_ dimer. Another factor influencing the value of this work can be the reverse i-motif orientation on the GR-NH_2_ surface.

**Figure 16:**
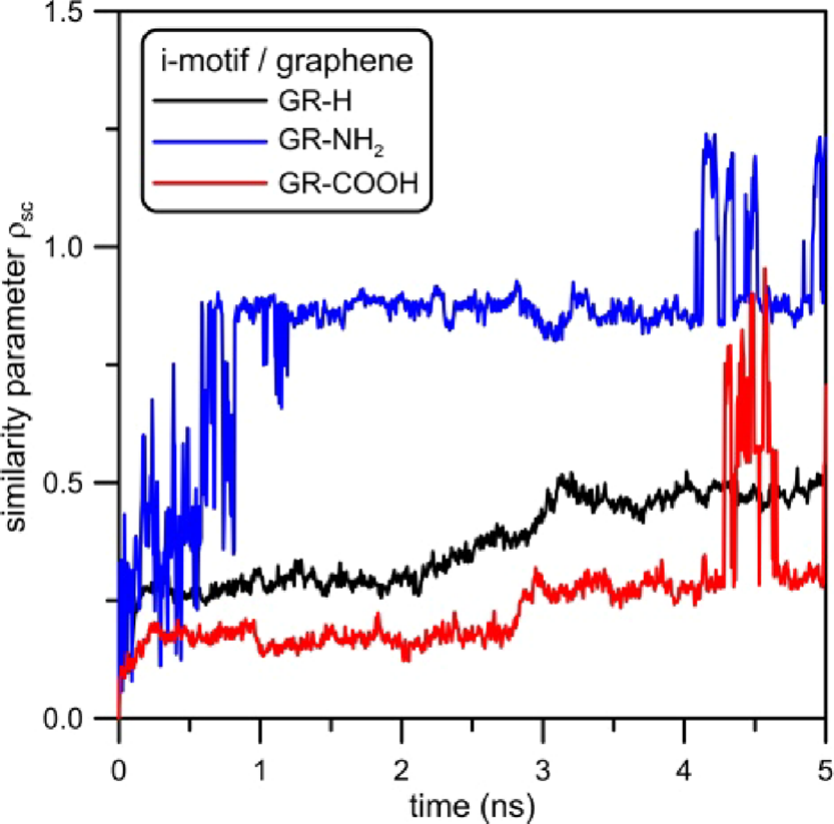
Size-independent scaled similarity parameter ρ_sc_ of the i-motif plotted as the function of dragging time from functionalized graphene surfaces. Reference states are the structures of i-motifs at the zeroth timestep of dragging, i.e., the states when DNA fragments are adsorbed on graphene surfaces and no force is applied to their COMs.

Regardless of the type of functionalized graphene, the time necessary to drag the DNA fragment out is about 3 ns. In the case of i-motif/GR-H and i-motif/GR-COOH, the extreme (initial and final) parts of work profiles are similar. Visible difference can be seen in the range of 1.0 to 3.5 ns. It suggests that the spring attached to the i-motif COM during biased simulations causes not only its desorption, but also influences the i-motif spatial structure. This is one of the reasons why calculated work is not the same as the free energy of interaction. To check it more carefully, in Figure 16, we show the comparison of the size-independent, scaled similarity parameters ρ_sc_ of the i-motif dragged out from graphene surfaces.^35^

As can be expected, the spatial structure of the i-motif is modified to the greatest extent in the case of dragging it from GR-NH_2_. In this case, up to 1 ns of simulation, the similarity parameter ρ_sc_ varies in the range of 0 to 0.7. Following Maiorov and Crippen,^35^ when ρ_sc_ > 0.5, it can be interpreted as antisimilarity from the initial structure. Next, when the DNA fragment is dragged out from GR-NH_2_ surface (times in the range of 1 to 4 ns), the similarity parameter remains constant. Finally, when the i-motif is desorbed, its structure randomly changes during some relaxations connected with shape optimization. In other cases, the similarity parameters ρ_sc_ of the i-motif adsorbed on GR-H and GR-COOH are much lower. Their values are in the range of 0.3 to 0.5 and can be interpreted as visually recognizable similarity of the i-motif structure to the reference state.^35^

**Figure 17:**
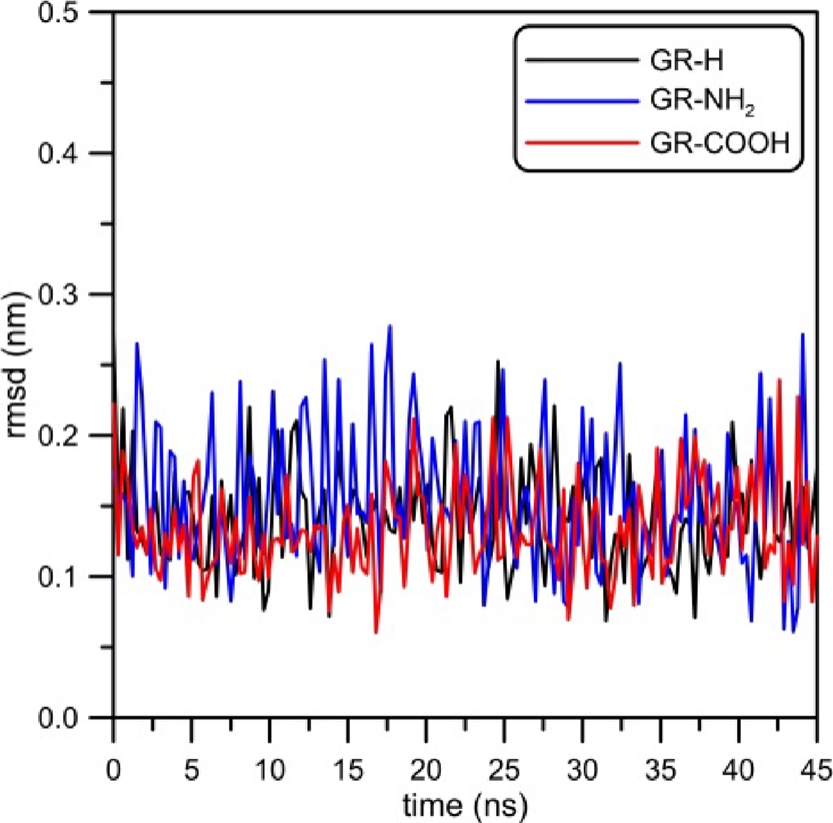
RMSD plotted as the function of time for GR-H, GR-NH_2_, and GR-COOH in the analyzed systems. Reference states are the structures of graphene at the zeroth timestep.

Although the main aim of the paper is the investigation of the changes in the spatial i-motif structure caused by adsorption, it is worth seeing the effect of DNA adsorption on the graphene sheet. Figure 17 shows the RMSD of graphene sheets plotted as a function of time. The curves were determined in the same way as in the case of plots presented in Figure 8.

It can be stated that the changes of RMSD are small and basically do not exceed 0.2 nm. This indicates a high degree of similarity of graphene sheets to their initial states when DNA was far from the surface. The changes in RMSD are caused by thermal fluctuations. It is worth noticing that RMSD plots shown in Figure 8 indicate the moment when the adsorption of the i-motif occurs. Simultaneously, the adsorption of DNA has no visible effect on the RMSD plots calculated for graphene structures.

## 4 Summary

The presented studies enable to recognize new physical insights into the interaction of the i-motif with functionalized graphene. First of all, the i-motif is very stably bonded to the graphene surface. The minimum distance between these two structures depends on the type of graphene functionalization, but the shortest one is in the case of amine-doped graphene. Further analysis showed that the i-motif/GR-NH_2_ system is much more different from the rest. In the case of i-motif adsorption on GR-H and GR-COOH, the DNA fragment oriented its 3′ and 5′ ends toward graphene, whereas on the GR-NH_2_ surface it is inversely oriented.

While considering the influence of graphene plane on the i-motif structure, our investigations suggest the changes of its internal structure due to adsorption interactions. Such changes consist of the stretching or shortening of the DNA chain as well as changing the distances between i-motif strands. These observations were confirmed by i-motif’s RMSD curves. The contact between the DNA fragment and graphene sheet always results in a change of the i-motif spatial structure. The analysis of fluctuations of i-motif atomic positions shows that the adsorption on graphene does not affect strongly bonded semiprotonated cytosine pairs. The fluctuations of 3′ thymine are large when it is adsorbed on GR-H and almost the same in other cases. When the DNA fragment is adsorbed on GR-COOH, a notable flexibility of thymine and adenines appears. The adsorption of the i-motif on GR-NH_2_ causes the stabilization of the upstream part of the DNA strand, and the mobility of thymine and adenines is very low. In the case of GR-H and GR-COOH, the opposite effect is observed.

The system i-motif/GR-NH_2_ is also distinguished from the others by the location of the adsorbed DNA fragment on the graphene surface. For almost all simulation trajectories, the i-motif is adsorbed very close to the amine-doped graphene edge. Moreover, only in the case of the i-motif/GR-NH_2_ dimer, the adsorption influences the internal H-bonds formed between nucleobases. These hydrogen bonds between nucleotides inside the i-motif structure have a strong character with the dominant of strongly covalent interactions.

The biased MD simulations confirmed the strongest i-motif adsorption on the amine-doped graphene. The work necessary to drag it out from the surface is almost twice larger than in other cases. Dragging DNA from the graphene surface causes significant changes in its shape. Thus, one of the most important conclusions is that the i-motif/GR-NH_2_ dimer is the most stable among the considered systems.

The observed features of investigated systems are very promising and encourage further experimental and theoretical research.

## Supporting Information Available

Short videos for all three cases showing the structural changes of the i-motif against the simulation time. Movies were made with visual MD software^36^ (MPG) (MPG) (MPG)

## Supplementary Material

jp2c04327_si_001.mpg

jp2c04327_si_002.mpg

jp2c04327_si_003.mpg
